# Cinteny: flexible analysis and visualization of synteny and genome rearrangements in multiple organisms

**DOI:** 10.1186/1471-2105-8-82

**Published:** 2007-03-08

**Authors:** Amit U Sinha, Jaroslaw Meller

**Affiliations:** 1 Department of Computer Science, University of Cincinnati, Cincinnati, OH 45221, USA; 2 Department of Environmental Health, University of Cincinnati College of Medicine, Cincinnati, OH 45267-0056, USA; 3 Department of Informatics, Nicholas Copernicus University, 87-100 Torun, Poland

## Abstract

**Background:**

Identifying syntenic regions, i.e., blocks of genes or other markers with evolutionary conserved order, and quantifying evolutionary relatedness between genomes in terms of chromosomal rearrangements is one of the central goals in comparative genomics. However, the analysis of synteny and the resulting assessment of genome rearrangements are sensitive to the choice of a number of arbitrary parameters that affect the detection of synteny blocks. In particular, the choice of a set of markers and the effect of different aggregation strategies, which enable coarse graining of synteny blocks and exclusion of micro-rearrangements, need to be assessed. Therefore, existing tools and resources that facilitate identification, visualization and analysis of synteny need to be further improved to provide a flexible platform for such analysis, especially in the context of multiple genomes.

**Results:**

We present a new tool, Cinteny, for fast identification and analysis of synteny with different sets of markers and various levels of coarse graining of syntenic blocks. Using Hannenhalli-Pevzner approach and its extensions, Cinteny also enables interactive determination of evolutionary relationships between genomes in terms of the number of rearrangements (the reversal distance). In particular, Cinteny provides: i) integration of synteny browsing with assessment of evolutionary distances for multiple genomes; ii) flexibility to adjust the parameters and re-compute the results on-the-fly; iii) ability to work with user provided data, such as orthologous genes, sequence tags or other conserved markers. In addition, Cinteny provides many annotated mammalian, invertebrate and fungal genomes that are pre-loaded and available for analysis at .

**Conclusion:**

Cinteny allows one to automatically compare multiple genomes and perform sensitivity analysis for synteny block detection and for the subsequent computation of reversal distances. Cinteny can also be used to interactively browse syntenic blocks conserved in multiple genomes, to facilitate genome annotation and validation of assemblies for newly sequenced genomes, and to construct and assess phylogenomic trees.

## Background

The increasing number of newly sequenced genomes is stimulating interest in comparative genomics and highlighting the need for the development of efficient tools that enable assessing their evolutionary relatedness. One problem of central importance is the identification of blocks of genes (or other markers) with evolutionary conserved order. These synteny blocks help in tracing back the evolution of genomes in terms of rearrangement events, such as reversals, translocation, fusion, fission etc. Consequently, genome evolution and phylogenetic (or phylogenomic) trees can be reconstructed from the analysis of synteny [1-5]. Furthermore, the presence of conserved blocks of genes in multiple genomes may also indicate functional relatedness of their products, e.g., in terms of protein-protein interactions [6] or presence of functionally important non-coding regions [7].

The evolutionary distance between two genomes can be expressed in terms of the minimum number of reversals to transform one genome into another, which is also referred to as the reversal distance [1-5]. In this approximation, translocation, fission and fusion events are represented as reversals, whereas the effect of transpositions is neglected. Furthermore, it is important to realize that RD is dependent on the choice of a set of discrete markers that are conserved between two (or more) genomes, the actual definition of synteny blocks (that may allow for small divergences in gene order, e.g. due to micro-rearrangements), the algorithm used to detect synteny blocks and various other parameters. Therefore, synteny identification and the resulting reversal distances need to be carefully assessed with respect to these choices, requiring fast algorithms and flexible tools.

A number of browsers, including Ensembl SyntenyView [9,10] and NCBI's MapViewer [11] can be used to visualize synteny for well annotated genomes. While the existing tools offer a variety of graphical representations of synteny blocks, they typically show pre-computed results for selected genomes and do not allow users to interactively assess the effects of the choice of a set of markers or other critical parameters, such as the minimum length of synteny blocks or the required minimum number of markers within a block. Furthermore, the evolutionary distances between genomes of different species are not computed directly by these tools. We developed a new web server, called Cinteny, which combines fast computation and assessment of the reversal distance with interactive synteny browsing and visualization in multiple genomes.

Thanks to remarkable algorithmic developments, the reversal distance (RD) can be efficiently computed in linear time [2,8]. Utilizing these algorithmic achievements, the GRIMM server was developed [12] to compute reversal distances for arbitrary pairs of genomes. However, the genomes of interest, as defined in terms of specific markers, must be first converted to signed numeric permutations by the user. This makes the use of GRIMM and interpretation of its results somewhat tedious, especially for multiple genomes. Cinteny addresses these limitations and additionally provides a number of features that facilitate identification and visualization of synteny blocks and measuring the reversal distance in multiple genomes.

## Implementation

### Data structure and algorithm

In order to efficiently represent the linear order of markers on a genome, Cinteny uses a tailored data-structure, which is implemented as a straightforward extension of ternary search trees (TST) [13]. In particular, we extended TSTs by "walks" through the leaves of the tree, which correspond to "walks" on the genome markers in their linear order. Such markers can be defined, e.g., by using the notion of orthologous genes, which are also referred to as homologous genes to indicate the similarity or homology between their sequences. Thus, homologous genes can be identified in the genomes of interest using sequence alignment and other annotation methods. We would like to comment, however, that the data structure and implementation considered here are, in fact, applicable to other readily available sets of unique markers that can be identified in the genomes of interest, as discussed in the Results and Discussion section.

**Figure 1:**
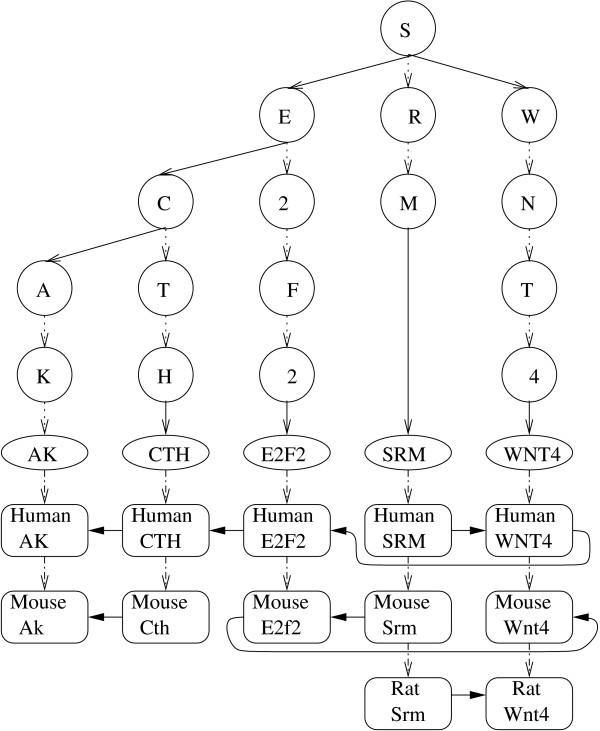
Ternary Search Tree-based representation of "genomic walks".

Figure 1 shows an example of a TST used by the Cinteny server, with several genes from the human, mouse and rat genomes as well as the corresponding walks. The TST is constructed with the gene symbols (nodes of the TST are represented by round circles in the figure, e.g., S, E, etc.) and the leaf nodes (shown as ovals, e.g., AK, CTH, etc.) represent each homologous group (a unique string representing the name of a gene representing that group). The individual genes belonging to each homologous group are connected below the leaf node as meta nodes (shown as rounded rectangles, Human AK, Mouse Ak, Human CTH, etc.). Linear walks are formed by connecting meta nodes based on the order in which the markers appear on a chromosome, as shown by arrows connecting the meta nodes, e.g., *Mouse Srm *→ *Mouse E2f2 *→ *Mouse Wnt4*. The strandedness (orientation) of a gene is stored in the node using an additional variable, and thus the whole genome may be formally represented as a signed permutation. The latter is required in order to apply the Hannenhalli-Pevzner theorem and based on it efficient algorithmic solutions [2].

There are several advantages of this hybrid data-structure: i) a gene may be searched in logarithmic time as the complexity of TST is similar to a binary search tree; ii) starting with a gene, one can directly enumerate all orthologs; iii) starting with a gene, one can visit the genes upstream and downstream by traversing up or down the walk. These factors facilitate the identification of conserved blocks and their representation in the form of a signed numeric permutation in linear time. Consequently, the reversal distance can be computed in linear time overall, using the approach by Hannenhalli and Pevzner [2], with modifications proposed by Bader and colleagues [8]. Moreover, the construction of the TST graph and associated walks scales linearly in practice (with the theoretical complexity of *N*log(*N*) where *N *is the number of markers), which makes it is suitable for large genomes. As result, a typical query, involving computation of the reversal distance for a pair of mammalian genomes, takes only a few CPU second on a typical desktop PC.

Initially, we define the synteny blocks by identifying blocks of conserved markers without any disruption of order and all the genes or other markers having the same relative orientation, i.e., two signed permutations representing these blocks being identical up to one reversal operation. These initial blocks with perfectly conserved order are subsequently extended (aggregated) into larger (non-overlapping) synteny blocks by ignoring smaller blocks, such as those resulting from micro-rearrangements. The aggregation strategy used here is based on that of Peng and colleagues [14] and involves adjustable parameters defined in the Results and discussion section (e.g., minimum length of synteny blocks).

These parameters effectively define the extent to which small divergences are tolerated within extended synteny blocks. We note also that many divergences in gene (marker) order may involve markers only observed in some genomes; these are thus automatically filtered out when multiple genomes are used, as described in the next section. The resulting coarse-graining leads to further computational simplifications, making Cinteny suitable for large scale comparisons of multiple genomes and a comprehensive sensitivity analysis of the results.

### Using multiple genomes

Finding synteny blocks and the reversal distance typically involves identifying markers (genes, sequence tags, etc.) that are shared by two species of interest. For example, the synteny between human and mouse can be analyzed using 15,645 orthologs, as identified by HomoloGene [11]. Using Cinteny, one may extend this approach to include a reduced set of markers common to multiple species, e.g., 6,425 genes common to human, chimpanzee, dog, mouse and rat genomes. In the latter case, the pair wise synteny (and reversal distance) between human and mouse may be found using this reduced set of 6,425 genes (markers). The advantage of this approach is that only highly conserved segments are used and the aggregation of synteny blocks is taken care of automatically, filtering out micro-rearrangements. On the other hand, in some cases this strategy may lead to a significant loss of data, especially if one of the genomes included in the analysis is poorly annotated, resulting in many missing genes or other markers. We stress, however, that the multiple genome-based approach is just one of several available options for synteny block detection that can now be easily compared with alternative strategies.

### Visualization

Cinteny enables analyses of genome rearrangements at three different levels: genome-wide, chromosome or around individual genes. The graphical layer for synteny browsing and analysis is built using PHP and enables intuitive interpretation of the results. All graphical representations are cross-linked and can be browsed interactively. For example, one can easily jump from a genome level view to a chromosome level view, or further magnify a synteny block for a detailed study. The graphical elements are also linked to external resources, such as NCBI. Public domain packages, including GBrowse [15] and SynView [16] are also used to provide alternative views of synteny blocks.

## Results and Discussion

In this section, we demonstrate examples of interrogating evolutionary relatedness of genomes using the Cinteny server. Different types of queries and different setup of parameters are illustrated. In particular, we show how the Cinteny web server can be used to identify synteny blocks and compute the reversal distance (RD) between whole genomes as well as between any two chromosomes of two genomes.

In order to perform sensitivity analysis for the computation of RD and identification of synteny blocks, Cinteny allows the user to interactively adjust several parameters, including: i) minimum length of synteny blocks (denoted as min_len); ii) maximum gap between adjacent blocks for aggregation (denoted as max_gap); iii) minimum number of markers in a block (denoted as min_num). Aggregation refers here to combining smaller synteny blocks to form larger blocks, wherever feasible. For example, by increasing the length between adjacent blocks (max_gap), one may effectively join segments which are otherwise far apart and obtain longer synteny blocks. Thus, such aggregation provides an effective coarse-graining of synteny blocks and affects the resulting RDs.

Another problem in applying algorithms for synteny block identification is posed by degenerate markers, such as paralogs (i.e., multiple copies of the same gene). In particular, the Hannenhalli-Pevzner theorem assumes that markers are unique [2]. In order to address this problem, Cinteny offers several different options for dealing with paralogs and enabling the assessment of these heuristics. One rational strategy is to use a paralog which lies within the most conserved region (i.e., the largest synteny block). In fact, this is the default option used also for all the examples shown in this paper. Other options, which are provided to enable an assessment of the results of such arbitrary choices, include the use of a random paralog or ignoring all genes which have paralogs.

### Whole genome analysis

**Figure 2:**
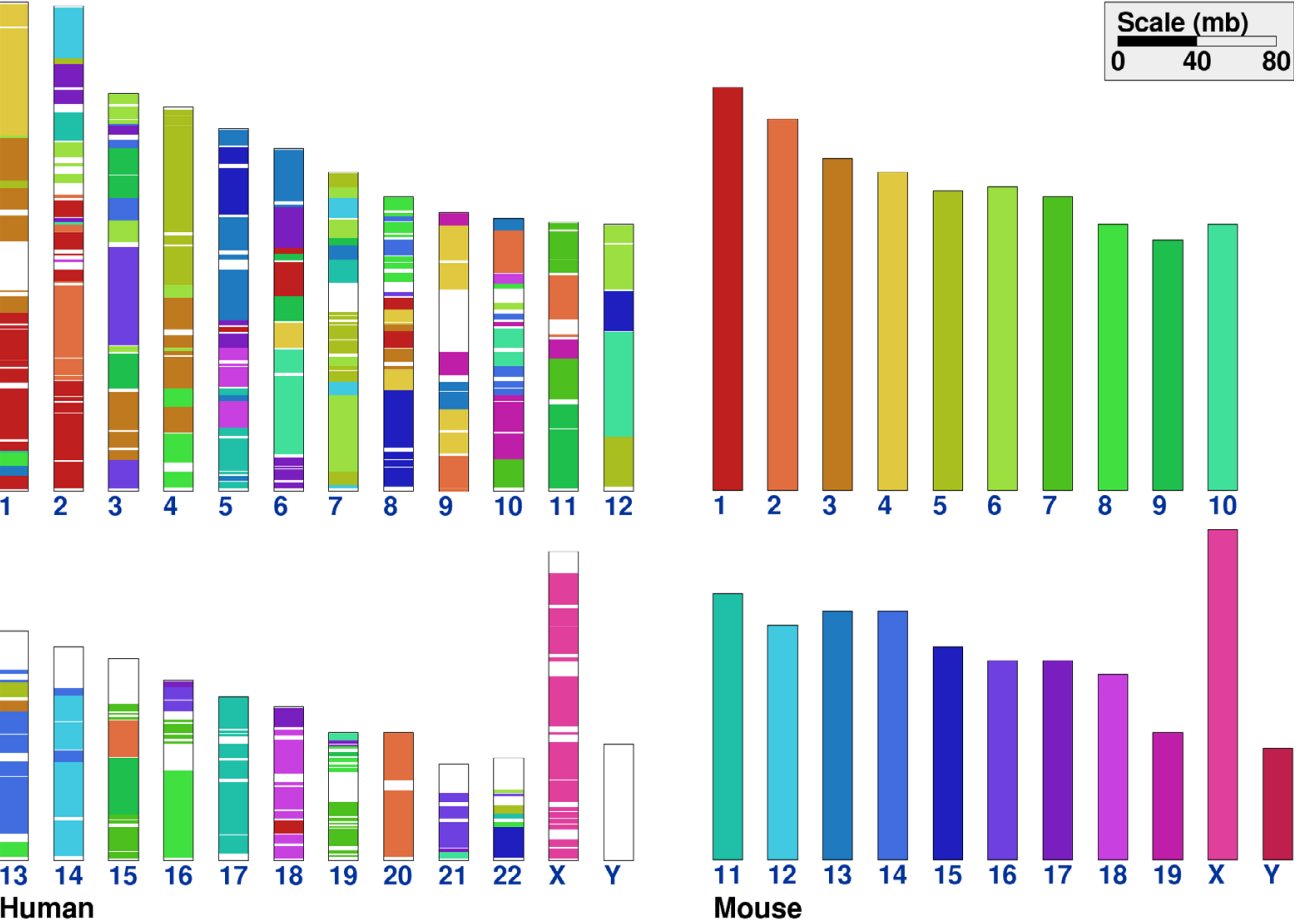
Visualization of synteny between human and mouse genomes.

Finding synteny and the RD between whole genomes is discussed here as an example of a typical application in comparative genomics. Figure 2 shows the synteny blocks for human and mouse genomes. The genome shown in the right panel (mouse in this case) is the source genome and the genome on the left (human) is the target genome. All chromosomes of the source genome are shown in unique colors. Each chromosome of the target genome is shown as composed of segments of some chromosome of the source genome, as indicated by the corresponding color. For example, the majority of human chromosome 1 is composed of (i.e. is syntenic to) mouse chromosomes 4, 3 and 1.

The figure was generated using a particular level of coarse-graining (aggregation), as defined by min_len = 300 Kb, max_gap = 1 Mb and min_num = 3. The number of synteny blocks found with these parameters and using the set of 15,645 human-mouse orthologs, as identified by HomoloGene 46.1 [11], is 359 and the RD is 261. These results are in qualitative agreement with previous studies [17]. However, it should be noted that by changing the level of coarse graining one may obtain very different results. For example, using min_len = 0, max_gap = 0 and min_num = 2 (this experiment is equivalent to no aggregation of synteny blocks) we find that number of synteny blocks increases to 828 and the RD to 348, respectively. We discuss further the problem of the dependence of the results on the parameters in Multiple Genomes section.

### Chromosome level analysis

**Figure 3:**
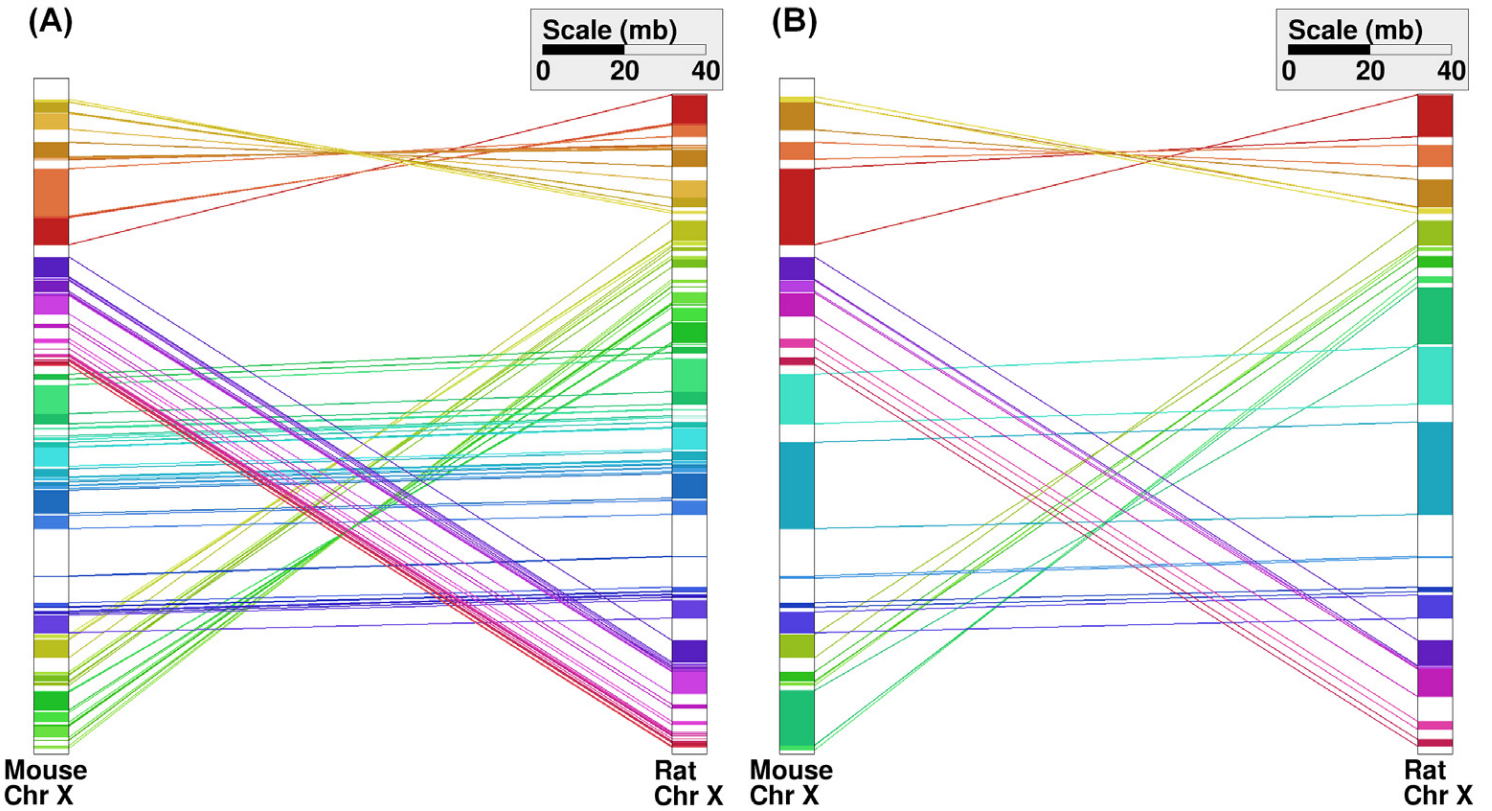
Comparison of X chromosomes of mouse and rat with (Panel B) and without (Panel A) aggregation.

Cinteny can also be used to identify synteny blocks and reversal distance between two chromosomes. In this case we use common markers located on the chromosomes of interest. In Figure 3, we show an example of synteny for the X chromosome of the mouse and the rat genomes. Figure 3A shows the syntenic blocks obtained without any aggregation (except for imposing that min_num = 2). The number of synteny blocks is 85 and the RD is 52 in this case. On the other hand, when using the aggregation described in the previous section, the number of synteny blocks is reduced to 17 and the RD to 8, respectively (see Figure 3B). As discussed later, similar results were obtained by imposing natural coarse-graining that utilizes multiple genomes.

### Analysis of individual synteny blocks

**Figure 4:**
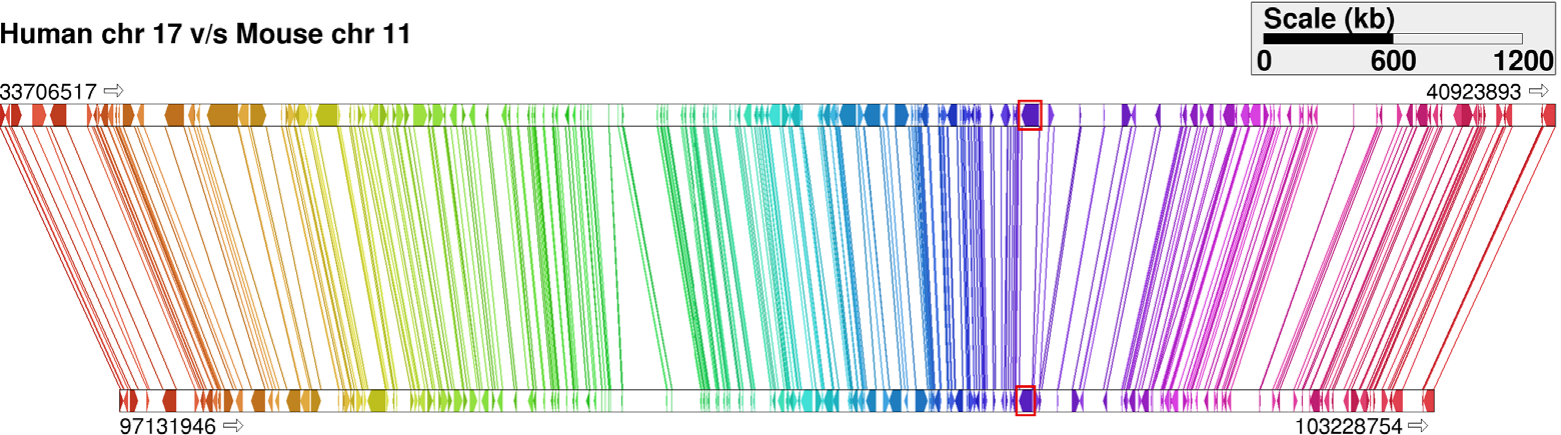
Synteny block view: human chromosome 17 (top) and mouse chromosome 11 (bottom) segments which contain the gene BRCA1.

In addition to queries illustrated above, one may use Cinteny for visualization and analysis of the synteny around a specific marker or gene. For example, starting with human and mouse genomes and default aggregation parameters, we find that the human BRCA1 gene is present in a conserved region of size 7.2 Mb in human chromosome 17, whereas its mouse ortholog (Brca1) is present in a conserved region of size 6.1 Mb on mouse chromosome 11 (see Figure 4). On the other hand, the DKK1 gene is present in a conserved region of size 2 Mb in the human genome and its ortholog, Dkk1, is present in a conserved region of size 1.7 Mb in the mouse genome. Several examples of such queries are available in the online help available at the Cinteny web site.

### Using multiple genomes

**Figure 5:**
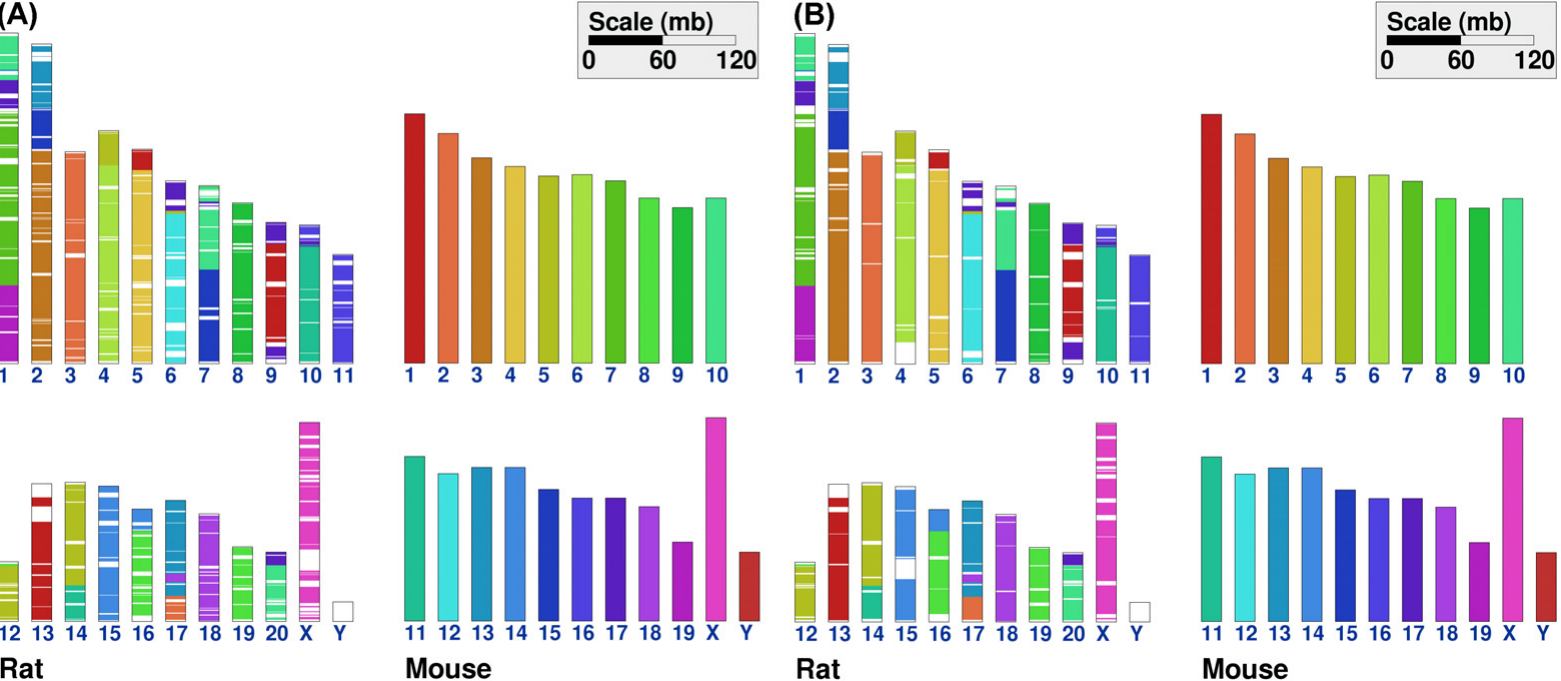
Comparison of whole genome synteny between rat and mouse using two-way (Panel A) and multi-way (Panel B) approach.

The use of multiple genomes for genome rearrangement analysis has been proposed before, e.g., in order to derive relationships between canine and other mammalian genomes [18]. Cinteny web server allows one to perform a 2-way (two genome-based) as well as multi-way (multiple genome-based) analysis. As an example, Figure 5 compares the whole genome synteny between rat and mouse genomes, as identified using 2-way and 5-way strategies that utilize subset of markers common to two (rat and mouse) or five genomes (human, mouse, dog, chimpanzee and rat), respectively (see also the Implementation section).

An intermediate aggregation level is used here for the two-way comparison (Figure 5A), with min_len = 100 kb and max_gap = 100 kb, leading to a reversal distance of 128. In Figure 5B, the same setup of parameters is used, except that only orthologs common to the five mammalian genomes are used. In the latter case, the RD of 86 is obtained. In addition, one can see that there are, in general, fewer gaps (represented by white spaces) in the 5-way analysis (Figure 5B), as a result of a natural coarse-graining due to selecting only highly conserved markers included in 5-way analysis.

Thus, as illustrated above, the absolute values of reversal distances may vary significantly with different choices of markers and aggregation strategies. We would like to comment, however, that RD is much more sensitive to the choice of parameters (min_len in particular) when using the 2-way approach, which can be easily verified using the Cinteny server. It also interesting to note that in relative terms (e.g., when using the mouse to rat distance normalized by the human to mouse distance) the reversal distances appear to be quite constant for a range of aggregation parameters, especially when using the multiple genome approach (data not shown). This may suggest that RDs can be used to indicate evolutionary relatedness in relative terms, as long as proper parametrization of the problem is used. This is the subject of a future work.

To further illustrate the usefulness of multi-way approach, we performed a 5-way analysis for the X chromosome of mouse and rat without any aggregation, yielding the RD of 14 and 19 synteny blocks. Comparison with Figure 3B, as well as comparison of the number of synteny blocks and RDs obtained using different aggregation strategies, suggests that using multiple genome approach provides a natural coarse-graining that allows one to select appropriate aggregation parameters for genomes of interest. Additional examples, e.g., regarding fungal genomes that are characterized by very different gene densities and high levels of genome rearrangements (making the choice of suitable aggregation parameters even more difficult), are included in the on-line help. We also comment that recent efforts to better and more fully annotate orthologous genes in hundreds of sequenced genomes [19] will likely make tools for multiple genome-based analyses even more important.

## Conclusion

The identification of synteny blocks, and the subsequent calculation of the reversal distance, is highly sensitive to the choice of parameters. Therefore, sensitivity analyses and careful assessments of the choice of critical parameters are important for drawing meaningful conclusions from inter-genomic comparisons. We present a new tool which offers a flexible platform for such analysis, enabling customization of synteny block detection and sensitivity analysis for the resulting estimates of evolutionary relatedness and plausible scenarios of genome rearrangements from ancestral genomes.

Cinteny performs all computations on the fly, allowing the parameters to be adjusted and the results recomputed interactively. Since the computation of the reversal distance for a pair of mammalian genomes takes only about a few seconds, one can easily assess the effects of various approximations and different levels of coarse-graining. For example, blocks larger than 300 kb are typically used for analysis of mammalian genomes. However, a different threshold may be more appropriate for smaller genomes, e.g., fungal genomes. In particular, a natural aggregation of synteny blocks can be achieved using multiple genomes. In addition, the effect of paralogs may be assessed by choosing a range of options, from removing all paralogs to using the ones that are contained within the largest conserved blocks.

While some well-annotated genomes like human, mouse, rat, dog, chimpanzee, nematode, drosophila, fungal genomes, etc. are pre-loaded, Cinteny also allows users to upload their own data. Hence, it can readily be used for the analysis of newly sequenced genomes, with user provided set of markers. Automated identification of conserved sequenced tags for multiple, unannotated (e.g., newly sequenced) genomes is being implemented as an extension of the current work.

## Availability and requirements

• Project Name: Cinteny

• Project home page: 

• Operating System(s): Platform independent (Web-based)

• Programming languages: C++ and PHP

• Other Requirements: None

• License: GNU GPL

• Restriction to use by non-academics: license needed.

## List of abbreviations

RD – Reversal Distance

## Authors' contributions

JM conceived of the study. AUS and JM designed the software and AUS implemented it. AUS and JM drafted the manuscript. All authors read and approved the final manuscript.
